# HMGB1 overexpression as a prognostic factor for survival in cancer: a meta-analysis and systematic review

**DOI:** 10.18632/oncotarget.10413

**Published:** 2016-07-06

**Authors:** Tengyun Wu, Wei Zhang, Geliang Yang, Huijun Li, Qi Chen, Ruixiang Song, Lin Zhao

**Affiliations:** ^1^ Air Force General Hospital of Chinese People's Liberation Army, Beijing 100142, China; ^2^ Department of Urology, Changhai Hospital, Second Military Medical University, Shanghai 200433, China; ^3^ Department of Integrated Oncology, Changhai Hospital, Second Military Medical University, Shanghai 200433, China; ^4^ The Wright Center, Scranton, Pennsylvania 18510, USA; ^5^ Department of Health Statistics, Faculty of Health Service, Second Military Medical University, Shanghai 200433, China

**Keywords:** high mobility group box 1, cancer, survival, prognosis factor, meta-analysis

## Abstract

As there are millions of cancer deaths every year, it is of great value to identify applicable prognostic biomarkers. As an important alarm, the prognostic role of high mobility group box 1 (HMGB1) in cancer remains controversial. We aim to assess the association of HMGB1 expression with prognosis in cancer patients. Systematic literature searches of PubMed, Embase and Web of Science databases were performed for eligible studies of HMGB1 as prognostic factor in cancer. Pooled hazard ratios (HRs) and 95% confidence intervals (CIs) were calculated to evaluate the influence of HMGB1 expression on overall survival (OS) and progression-free survival (PFS) in cancer patients. 18 studies involving 11 different tumor types were included in meta-analysis. HMGB1 overexpression was significantly associated with poorer OS (HR: 1.99; 95% CI, 1.71-2.31) and PFS (HR: 2.26; 95% CI, 1.65-3.10) irrespective of cancer types including gastric cancer, colorectal cancer, hepatocellular carcinoma, pancreatic cancer, nasopharyngeal carcinoma, head and neck squamous-cell carcinoma, esophageal cancer, malignant pleural mesothelioma, bladder cancer, prostate cancer, and cervical carcinoma. Subgroup analyses indicated geographical area and size of studies did not affect the prognostic effects of HMGB1 for OS. Morever, HMGB1 overexpression had a consistent correlation with poorer OS when detected by immunohistochemistry in tissues and enzyme-linked immunosorbent assay in serum, whereas the correlation did not exist by quantitative real-time reverse-transcription polymerase chain reaction in tissues. HMGB1 overexpression is associated with poorer prognosis in patients with various types of cancer, suggesting that it is a prognostic factor and potential biomarker for survival in cancer.

## INTRODUCTION

According to the reports from WHO, there were 14.1 million new cancer cases, 8.2 million cancer deaths and 32.6 million people living with cancer (within 5 years of diagnosis) in 2012 worldwide [1]. Combination of surgery, radiotherapy and chemotherapy remains the standard treatment in most cancer cases, however, not all patients derive benefit from it [2]. In addition, more and more targeted agents and biotherapies are now available while the applicable patients are limited [3]. Therefore, it is critical to identify applicable prognostic biomarkers, guiding individualized treatment and improving unfavorable prognosis.

High mobility group box 1 (HMGB1) protein, which was discovered in calf thymus in 1973 [4], is a ubiquitous chromatin component expressed in nucleated mammalian cells. In 1990s', Wang's work demonstrated that HMGB1 is involved in the pathological process of sepsis for the first time [5]. Now we have known that HMGB1 was involved in transcription regulation of many cancer genes, including E-selectin, TNF-α, BRCA1 and insulin receptor [6–9]. In addition to these reports, recent evidence demonstrates that HMGB1 plays an important role in the tumorigenesis and progression of many types of cancers such as digestive system, urogenital system, skin, bone, and blood cancer [10–12]. However, HMGB1 acts as both a tumor suppressor and an oncogenic factor in tumorigenesis and cancer therapy depending on the context and HMGB1 location and modification [13]. The prognostic value of HMGB1 overexpression for survival across different tumors still remains controversial. Therefore, we performed a literature-based systematic review and meta-analysis in order to assess the association of HMGB1 expression with prognosis in patients with cancer.

## RESULTS

### Study characteristics

**Figure 1:**
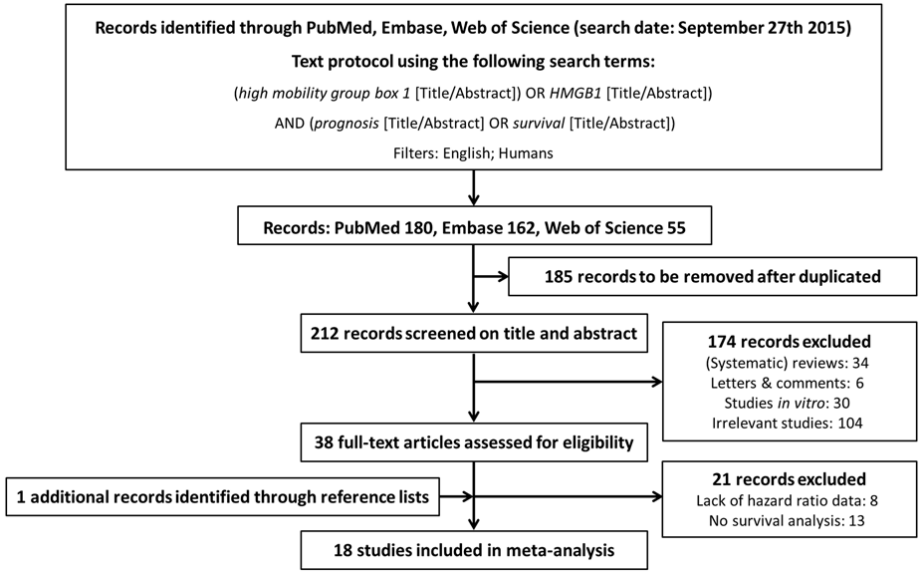
Flow diagram of search strategy

18 studies met our inclusion criteria and were finally included for the analysis, involving 11 different tumor types (3 studies of gastric cancer [14–16], 4 of colorectal cancer [17–20], 2 of hepatocellular carcinoma [21, 22], 2 of pancreatic cancer [23, 24], 1 of nasopharyngeal carcinoma [25], 1 of head and neck squamous-cell carcinoma [26], 1 of esophageal cancer [27], 1 of malignant pleural mesothelioma [28], 1 of bladder cancer [29], 1 of prostate cancer [30], and 1 of cervical carcinoma [31]) (Figure 1). A total of 2249 participants were analyzed for the association between HMGB1 expression and disease prognosis, of which 2090 (92.9%) and 1247 (55.4%) ones were respectively included into overall survival (OS) and progression-free survival (PFS) analyses. The detailed information of included studies was summarized in Table 1.

**Table 1:** Summary of 11 types of cancer studies included in the meta-analysis

Cancer type	Study	Institution (Country)	Study period	Specimen source	Detection method	Cases, n	
						HMGB1 high/ positive	HMGB1 low/ negative
gastric cancer	Akaike et al	University of Yamanashi (Japan)	1997-1998	tissue	IHC	35	41
	He et al	The first Affiliated Hospital of Sun Yat-Sen University (China)	2003-2005	tissue	IHC	56	83
	Zhang et al	Shanghai Sixth People's Hospital, Shanghai Jiaotong University (China)	2007-2011	tissue	IHC	32	18
	Yao et al	The First Affiliated Hospital of Jilin University (China)	2002-2006	tissue	IHC	107	85
colorectal cancer	Peng et al	Cancer Center of Sun Yat-Sen University (China)	1999-2002	tissue	IHC	40	32
	Soldevilla et al	Puerta de Hierro Majadahonda University Hospital (Spain)	2001-2003	tissue	qRT-PCR	51	26
	Ueda et al	Kyushu University Beppu Hospital (Japan)	1992-2002	tissue	qRT-PCR	70	70
hepatocellular carcinoma	Liu et al	Cancer Center of Sun Yat-Sen University (China)	2004-2005	tissue	IHC	69	92
	Xiao et al	General Hospital of Guangzhou Military Command of PLA (China)	1999-2002	tissue	IHC	134	74
pancreatic cancer	Chung et al	Yonsei University College of Medicine (Korea)	2006-2011	serum	ELISA	33	12
	Wittwer et al	University Hospital Munich-Grosshadern (Germany)	2006-2010	serum	ELISA	NA	NA
nasopharyngeal carcinoma	Wu et al	Nanfang Hospital, Southern Medical University (China)	1998-2002	tissue	IHC	89	77
squamous-cell carcinoma of the head and neck	Liu et al	Xiangya Hospital, Central South University (China)	2002-2004	tissue	IHC	45	58
esophageal cancer	Chuangui et al	Tianjin Medical University Cancer Institute and Hospital (China)	2001-2003	tissue	IHC	50	22
malignant pleural mesothelioma	Tabata et al	Hyogo College of Medicine Hospital (Japan)	2005-2009	serum	ELISA	NA	NA
bladder cancer	Yang et al	Renji Hospital, Shanghai Jiao Tong University (China)	2003-2005	tissue	IHC	87	77
prostate cancer	Li et al	Tongji Hospital, Tongji University (China)	2002-2010	tissue	IHC	95	64
cervical carcinoma	Sheng et al	Shandong Cancer Hospital and Institution (China)	2000-2008	serum	ELISA	NA	NA

IHC: immunohistochemistry; qRT-PCR: quantitative real-time polymerase chain reaction; ELISA: enzyme-linked immunosorbent assay; HMGB1: high-mobility group box 1.

Among 18 studies, 15 were prospective cohort researches (level of evidence: 1b) whereas 3 were retrospective designs (level of evidence: 2b). The methodological quality of included studies was relatively high for 17 cohorts (NOS: 7 of 9 points and 6 of 9 points) and medium for one (NOS: 5 of 9 points), which was mainly attributed to the lack of definite follow-up period and lost rate. According to the guidelines for assessing quality in prognostic studies, the evaluation results of each item with potential bias were presented as “yes”, “partly”, “no” or “unsure” in Table 2. The key baseline characteristics of patients were adequately presented and the adopted statistical analyses were appropriate in all included studies. However, study by Soldevilla et al [19] did not give a well-defined interpretation standard of HMGB1 expression. In addition, the duration of follow-up was not distinctly described in 4 studies of Akaike et al [14], He et al [15], Wittwer et al [24] and Sheng et al [31]. Unfortunately, there was no study mentioning important confounders like the subsequent treatment. The evaluation standards of HMGB1 expression and group definitions of HMGB1 high/positive or low/negative in included studies were summarized in Supplementary Table S1.

**Table 2:** Quality assessment of the studies included in the meta-analysis

Study	Quality evaluation of prognosis study						Study quality	Level of evidence
	Study participation	Study attrition	Prognostic factor measurement	Outcome measurement	Confounding measurement and account	Analysis		
Akaike et al	Yes	Yes	Yes	Partly	Partly	Yes	6	1b
He et al	Yes	Yes	Yes	Partly	Partly	Yes	6	1b
Zhang et al	Yes	Yes	Yes	Yes	Partly	Yes	7	1b
Yao et al	Yes	Yes	Yes	Yes	Partly	Yes	7	1b
Peng et al	Yes	Yes	Yes	Yes	Partly	Yes	7	1b
Soldevilla et al	Yes	Yes	Partly	Yes	Partly	Yes	7	1b
Ueda et al	Yes	Yes	Yes	Yes	Partly	Yes	6	1b
Liu et al	Yes	Yes	Yes	Yes	Partly	Yes	7	1b
Xiao et al	Yes	Yes	Yes	Yes	Partly	Yes	6	1b
Chung et al	Yes	Yes	Yes	Yes	Partly	Yes	7	1b
Wittwer et al	Yes	Yes	Yes	Partly	Partly	Yes	6	1b
Wu et al	Yes	Yes	Yes	Yes	Partly	Yes	7	1b
Liu et al	Yes	Yes	Yes	Yes	Partly	Yes	7	2b
Chuangui et al	Yes	Yes	Yes	Yes	Partly	Yes	5	2b
Tabata et al	Yes	Yes	Yes	Yes	Partly	Yes	7	1b
Yang et al	Yes	Yes	Yes	Yes	Partly	Yes	6	2b
Li et al	Yes	Yes	Yes	Yes	Partly	Yes	7	1b
Sheng et al	Yes	Yes	Yes	Partly	Partly	Yes	6	1b

### HMGB1 and OS

**Figure 2:**
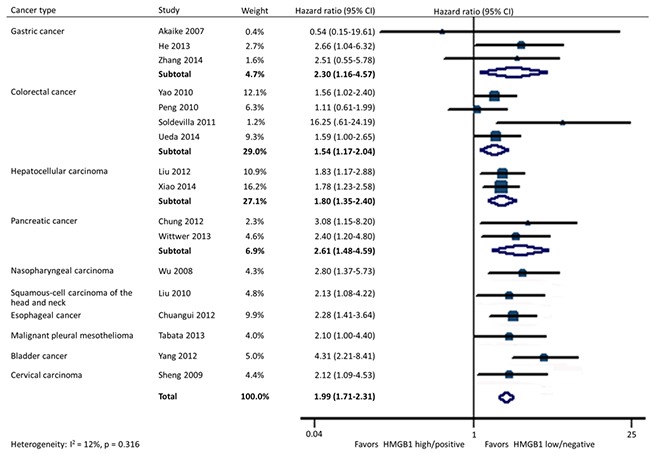
Forest plot evaluating association of HMGB1 expression and overall survival

In all, 17 studies included data on OS in 10 types of cancer. Homogeneity tests showed evidence of non-significant heterogeneity among studies in all the OS analyses except for the subgroup detected by quantitative real-time reverse-transcription polymerase chain reaction (qRT-PCR) in tissues. HMGB1 overexpression was significantly associated with a poorer OS in patients with cancer (HR: 1.99; 95% CI, 1.71-2.31). Furthermore, the statistical significance was constant irrespective of different tumor types containing gastric cancer (HR: 2.30; 95% CI, 1.16-4.57), colorectal cancer (HR: 1.54; 95% CI, 1.17-2.04), hepatocellular carcinoma (HR: 1.80; 95% CI, 1.35-2.40), pancreatic cancer (HR: 2.61; 95% CI, 1.48-4.59), nasopharyngeal carcinoma (HR: 2.80; 95% CI, 1.37-5.73), head and neck squamous-cell carcinoma (HR: 2.13; 95% CI, 1.08-4.22), esophageal cancer (HR: 2.28; 95% CI, 1.41-3.64), malignant pleural mesothelioma (HR: 2.10; 95% CI, 1.00-4.40), bladder cancer (HR: 4.31; 95% CI, 2.21-8.41), and cervical carcinoma (HR: 2.12; 95% CI, 1.09-4.53) (Figure 2).

**Figure 3:**
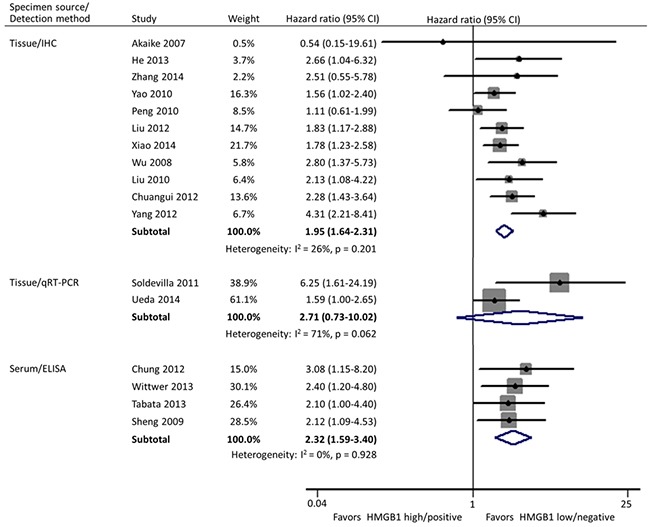
Subgroup forest plot evaluating association of HMGB1 expression and overall survival by specimen source and detection method

**Figure 4:**
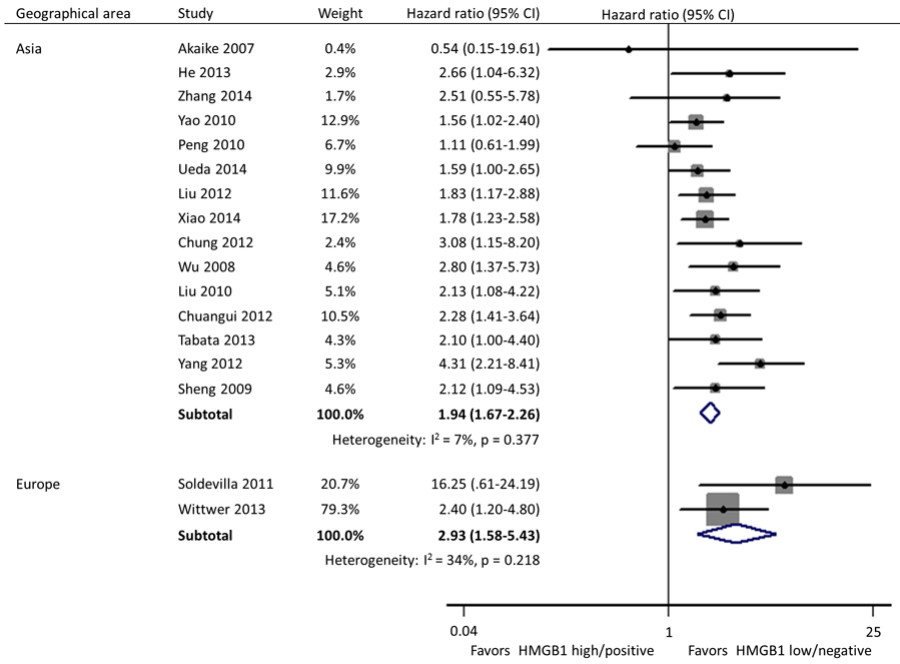
Subgroup forest plot evaluating association of HMGB1 expression and overall survival by geographical area

**Figure 5:**
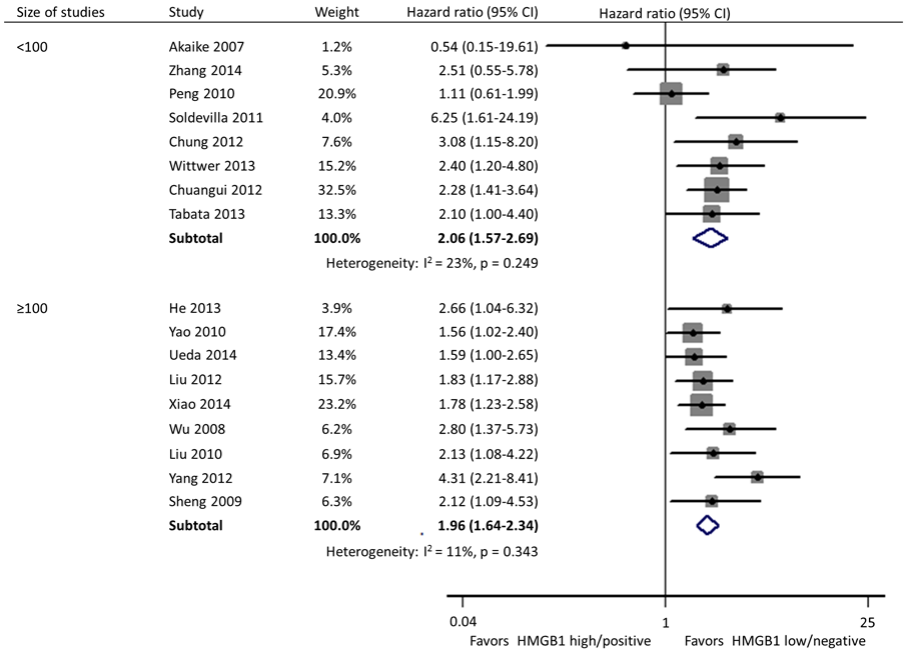
Subgroup forest plot evaluating association of HMGB1 expression and overall survival by size of studies

Subgroup analyses were carried out according to specimen source and detection method, geographical area, and size of studies. In these 17 studies, HMGB1 was respectively detected using immunohistochemistry (IHC) staining and qRT-PCR in cancer tissues by 11 and 2 researches, and the remaining 4 investigations evaluated the expression of serum HMGB1 using enzyme-linked immunosorbent assay (ELISA). Different from the other two practice of IHC in tissues (HR: 1.95; 95% CI, 1.64-2.31) and ELISA in serum (HR: 2.32; 95% CI, 1.59-3.40), HMGB1 expression had no correlation with the OS of patients when detected by qRT-PCR in tissues (HR: 2.71; 95% CI, 0.73-10.02) (Figure 3). Geographically, 15 studies were conducted in Asia (11 in China, 3 in Japan and 1 in Korea) and the remaining 2 in Europe (1 in Spain and 1 in Germany). The OS was significantly shorter in patients with high HMGB1 expression compared those with low HMGB1 expression in both Asia (HR: 1.94; 95% CI, 1.67-2.26) and Europe (HR: 2.93; 95% CI, 1.58-5.43) (Figure 4). The sample size of included studies ranged from 45 to 286, and 9 ones enrolled more than 100 participants each. Whatever, the HRs for OS were similar between the subgroups with <100 patients (HR: 2.06; 95% CI, 1.57-2.69) and ≥100 patients (HR: 1.96; 95% CI, 1.64-2.34) (Figure 5).

### HMGB1 and PFS

**Figure 6:**
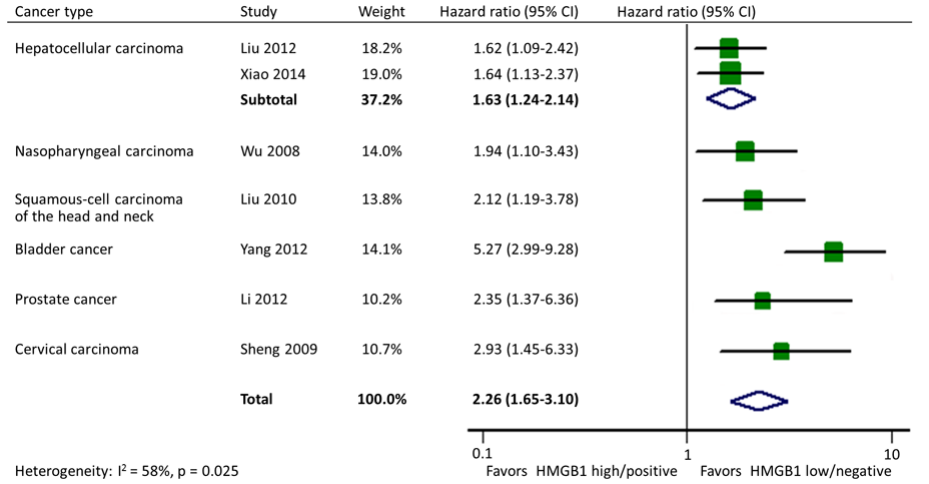
Forest plot evaluating association of HMGB1 expression and progression-free survival

There were 7 studies reporting data on PFS in 6 types of cancer altogether. Homogeneity test indicated the heterogeneity among studies so the random-effects model was used. The pooled estimate showed a significant shorter PFS in cancer patients with HMGB1 overexpression (HR: 2.26; 95% CI, 1.65-3.10). In addition, the effects of HMGB1 overexpression on PFS were consistent among different tumor types: hepatocellular carcinoma (HR: 1.63; 95% CI, 1.24-2.14), nasopharyngeal carcinoma (HR: 1.94; 95% CI, 1.10-3.43), head and neck squamous-cell carcinoma (HR: 2.12; 95% CI, 1.19-3.78), bladder cancer (HR: 5.27; 95% CI, 2.99-9.28), prostate cancer (HR: 2.35; 95% CI, 1.37-6.36), and cervical carcinoma (HR: 2.93; 95% CI, 1.45-6.33) (Figure 6).

## DISCUSSION

The traditional assessment of prognosis in patients with cancer is mostly based on the tumor type, pathological grading, and clinical stage. However, it has been demonstrated that other factors like molecular and cellular characteristics of primary tumor may improve our prognostic evaluation [32], and then guide the development of appropriate surgical treatment strategy as well as the choice of postoperative management. In this article, we conducted a meta-analysis to evaluate HMGB1 mRNA and protein expression levels in tissue or serum from patients with cancer. The results indicated that HMGB1 overexpression was associated with poorer prognosis in patients with various types of cancer.

The high mobility group (HMG) proteins, a group of non-histone nuclear proteins with high electrophoretic mobility, were first discovered in 1973. This protein family includes three super families termed HMGB, HMGN, and HMGA [4]. As the most abundant and well-studied HMG protein, HMGB1 is indicated to be associated with ten functional capabilities that drive tumor development and growth [33]. These cancer hallmarks include: sustainment of proliferative signaling; evasion of growth suppressors; avoidance of immune destruction; enablement of replicative immortality; tumor-promoting inflammation; activation of invasion and metastasis; induction of angiogenesis; genome instability and mutation; resistance to cell death; and deregulation of cellular energetics [34]. Meanwhile, higher HMGB1 expression was detected in late stage patients with hepatocellular carcinoma [35] and squamous-cell carcinoma of the head and neck [26]. In this respect, HMGB1 can be considered as an oncoprotein. However, as reviewed by Kang et al [13], HMGB1 can act as both a tumor suppressor and an oncogenic factor in tumorigenesis and cancer therapy according to different experiment conditions. The underlying mechanisms involved in the relationship between HMGB1 expression and prognosis in patients with cancer are uncertain.

In current meta-analysis, we revealed that HMGB1 overexpression was significantly associated with a poorer OS and a shorter PFS in cancer patients, moreover, the statistical significance was constant irrespective of different tumor types. In 16 included studies, multivariate analysis with the Cox's proportional hazards model was also performed to demonstrate that HMGB1 was an independent prognostic factor for patients with cancer. Besides the overall evaluation, subgroup analyses were done in respect of specimen source and detection method, geographical area, and size of studies. The association of HMGB1 overexpression with survivals persisted and remained statistically significant under all the classification criteria except when the detection method of qRT-PCR in tissues was adopted. On one hand, the limited available studies and their small sample size may contribute to this insignificant. On the other hand, the fact existing is that there is no internationally accepted and validated method for HMGB1 expression assessment so far. Further researches are urged to determine the best detection procedure and then establish the consistent standard.

HMGB1 plays a crucial role not only inside the cell as a chromatin structural protein, but also outside the cell as a cytokine [36]. It can be secreted actively by various cells such as macrophages, monocytes, neutrophils and neurons [37]. In addition, damaged or necrotic cells can passively release HMGB1 protein as well [38]. Tumor cells overexpressing HMGB1 have been reported to secrete it to the extracellular matrix in erythroleukemia, neuroblastoma and colon cancer cells [39, 40]. This phenomenon usually occurs when tumor cells undergo necrosis or triggered by hypoxia, nutrient deprivation, absence of essential growth factors or application of conventional anticancer therapy [41, 42]. The released HMGB1 will trigger the chronic inflammatory response, promote tumor cell survival, invasion and neoangiogenesis through activation of intracellular signaling [43, 44]. As is known, the inflammatory tumor microenvironment can promote neoplastic transformation and support tumor growth, invasion and metastasis. Extracellular HMGB1, act as the prototypic damage associated molecular pattern molecule, can activate proinflammatory signaling pathways through conjunction with Toll-like receptors (TLRs) and receptor for advanced glycation end products (RAGE) to induce proinflammatory cytokine release. Inhibition of RAGE-HMGB1 interaction has been proved to be effective in inhibiting tumor angiogenesis and growth, metastasis, migration and invasion of cancer cells [45–47]. Therefore, serum HMGB1 can be a potential powerful diagnostic and prognostic biomarker for patients with cancer. Among included studies, 4 researches [23, 24, 28, 31] showed that overexpressed serum HMGB1 was closely correlated with the advanced stage of cancer in pancreatic cancer, cervical carcinoma, and malignant pleural mesothelioma. The pooled estimate of HR for subgroup detected by ELISA in serum in our meta-analysis further confirmed its prediction for progression of patients with cancer. Meanwhile, Chung et al [23] and Sheng et al [31] respectively exhibited serum HMGB1 was a remarkable biomarker to predict pancreatic ductal adenocarcinoma and recurrent cervical squamous cell carcinomas with superior sensitivity or specificity compared to existing biomarkers.

In addition to the included studies, there were some other researches focusing on the correlation between HMGB1 expression and patient prognosis in osteosarcoma [48], ovarian cancer [49] and non-small cell lung cancer [50]. As the definite HRs with 95% CIs were not described in these manuscripts, they were finally excluded from current meta-analysis. However, they also found that higher expression level of HMGB1 was significantly associated with a poorer prognosis. At the same time, Takeuchi et al [51] demonstrated that overexpression of RAGE, one of HMGB1′s receptors, was associated with subsequent recurrence, lung metastasis, and poor survival in chondrosarcoma. A recent meta-analysis also showed higher expression of C-X-C chemokine receptor 4 (CXCR4), another HMGB1′s receptor, indicated poorer prognosis in various types of cancer [2]. As a prognostic biomarker, we propose that HMGB1 has potential clinical applicability in certain aspects: (1) guide the therapy approaches selection and stratification; (2) monitor the response to a therapy for the decision whether it should be continued or not; (3) early alert to the possibility of cancer recurrence or metastasis. Furthermore, several recent researches discussed the combined application of HMGB1 with other existing biomarkers. HMGB1 has been demonstrated to work together with prostate specific antigen (PSA) for predicting biochemical recurrence in prostate cancer [20] or with squamous cell carcinoma antigen (SCCA) for early diagnosing recurrent cervical squamous cell carcinomas [32].

The identification of prognostic factors is critical for distinguishing high-risk patients who are good candidates for individualized treatment from others [52]. Our finding that HMGB1 overexpression is associated with poorer prognosis in cancer patients indicates this gene may have the potential to become a critical molecular target for tumor therapy. Jube et al [53] showed that treatment with HMGB1 inhibitors could prolong the survival of malignant mesothelioma xenograft mice, offering a preclinical proof-of-principle that antibody-mediated ablation of HMBG1 was sufficient to elicit antitumor therapeutic activity. At the same time, some studies have demonstrated that HMGB1 is constitutively expressed in the nucleus and perinuclear organelles of cancer cells with the active nucleo-cytoplasmic shuttling of HMGB1 existing [25]. As the functions of HMGB1 depend on the subcellular locations, its influence on tumor development and progression ought to be explained by different models. Future studies which focus on HMGB1 as a novel antitumor therapeutic approach should take it into consideration comprehensively.

Our study has several limitations. First, there were totally 2 specimen sources and 3 detection methods being involved within included studies. As a semi-quantitative measurement, IHC was adopted by majority of researches, however, only a few applied ELISA or qRT-PCR which were quantitative approaches in their studies. Among 11 studies using IHC, the cutoff values were based on the sum of the intensity and extent scores in 9 studies, whereas the cutoff points of rest 2 researches were solely determined by proportion of HMGB1 positive cells. Second, the HRs data of survival were extracted according to the univariate analysis in 2 included studies because the multivariate analysis was not performed. Third, there was a bias towards Asian patients because 11 of 15 studies were from China while only 3 from Japan and 1 from Korea. Lastly, the findings of this meta-analysis need to be confirmed by future complete and through studies in order to make a solid conclusion.

## MATERIALS AND METHODS

### Literature search and article selection

This meta-analysis was performed according to the Preferred Reporting Items for Systematic Reviews and Meta-Analyses (PRISMA) statement [54]. Literature searches of PubMed, Embase, and Web of Science databases were carried out in September 27th, 2015 using the following keywords: (high mobility group box 1 OR HMGB1) AND (prognosis OR survival). Search restrictions were set for the English language and human species. Electronic searches were supplemented by studying reference lists of the retrieved articles as well as relevant review articles. Each article was assessed for inclusion by T.W and W.Z independently and all disagreements were resolved via discussion.

### Inclusion and exclusion criteria

The inclusion criteria for the studies were as follows: (1) evaluating the association between HMGB1 expression and prognosis of patients with any type of cancer; (2) reporting endpoints including OS and PFS; (3) displaying outcomes in the form of hazard ratio (HR) with 95% confidence interval (CI). The exclusion criteria included: (1) duplicated studies using the same population or overlapping database; (2) non-human research or articles in non-English; (3) reviews, letters, and comments.

### Evaluation of study quality

The levels of evidence were estimated for all included studies with the Oxford Centre for Evidence Based Medicine criteria [55]. The methodological quality evaluation of the studies was conducted using the Newcastle-Ottawa Scale (NOS) for cohort study [56]. In addition, the specific quality assessment of prognosis studies was estimated according to the approach of Hayden et al [57]. The evaluated items with potential bias included study participation, study attrition, prognostic factor measurement, outcome measurement, confounding measurement and account, and analysis. The assessments were processed independently by two reviewers and the final decision was achieved by consensus.

### Statistical analysis

In order to pool the OS or PFS of various included studies, heterogeneity between studies was assessed using the Q and I^2^ statistics: Significant heterogeneity was defined as Q statistic p value <0.10 or I^2^ value >50%. A fixed-effects model was used if there was no evidence of heterogeneity; otherwise, a random-effects model was used. Meta-analysis was performed using the pooled HR with 95% CI as the risk estimate. An observed HR of >1 indicated poorer prognosis in patients with elevated HMGB1 expression. The HR was considered statistical superiority/inferiority between groups if the 95% CI did not overlap with 1. All p values were two-sided and statistical significance was set at p <0.05. Stata software version 12.0 (Stata Corporation, College Station, Texas, USA) was used to conduct statistical analysis.

## CONCLUSIONS

In summary, HMGB1 overexpression is associated with poorer prognosis in terms of OS and PFS in patients with various types of cancer, suggesting that it is a prognostic factor and potential biomarker for survival in cancer. Besides, HMGB1 presents as a potential antitumor target and further development of strategies against this receptor could be an attractive therapeutic approach.

## SUPPLEMENTARY MATERIALS TABLE


