# Supplementary material for: HMGB1 overexpression as a prognostic factor for survival in cancer: a meta-analysis and systematic review
Source: Oncotarget. 2016 Jul 6;7(31):50417–27. doi: 10.18632/oncotarget.10413 (PMC5226592; doi:10.18632/oncotarget.10413)
Supplement: Supplementary file 1 [file oncotarget-07-50417-s001.pdf]

HMGB1 overexpression as a prognostic factor for survival in cancer: a meta-analysis and systematic review

SUPPLEMENTARY TABLE

Supplementary Table S1: Summary of evaluation standards of HMGB1 expression in included studies

Study	Detection method	Definition of HMGB1 high/positive	Definition of HMGB1 low/negative	Evaluation standard of HMGB1 expression
Akaike et al	IHC	NA	NA	mean value of the counts of positive cancer cells
He et al	IHC	expression score ≥2	expression score <2	expression score (0-3)
Zhang et al	IHC	expression score ≥4	expression score <4	total expression score (0-12)
Yao et al	IHC	staining score ≥3	staining score <3	final staining score (0-7)
Peng et al	IHC	staining score >3	staining score ≤3	final staining score (0-7)
Soldevilla et al	qRT-PCR	NA	NA	NA
Ueda et al	qRT-PCR	NA	NA	median expression level in tumor tissues
Liu et al	IHC	multiply-indexes ≥9	multiply-indexes <9	multiply-indexes (1-16)
Xiao et al	IHC	staining score ≥3	staining score <3	final staining score (0-7)
Chung et al	ELISA	serum level >30ng/mL	serum level ≤30ng/mL	serum HMGB1 level
Wittwer et al	ELISA	serum level >2.4ng/mL	serum level <0.8ng/mL	serum HMGB1 level
Wu et al	IHC	staining score ≥3	staining score <3	final staining score (0-7)
Liu et al	IHC	staining score ≥6	staining score <6	final staining score (0-7)
Chuangui et al	IHC	staining score ≥3	staining score <3	final staining score (0-7)
Tabata et al	ELISA	serum level ≥9.0ng/mL	serum level <9.0ng/mL	serum HMGB1 level
Yang et al	IHC	staining score >3	staining score ≤3	final staining score (0-7)
Li et al	IHC	staining score ≥3	staining score <3	final staining score (0-7)
Sheng et al	ELISA	serum level >21.8ng/mL	serum level ≤21.8ng/mL	serum HMGB1 level

IHC: immunohistochemistry; qRT-PCR: quantitative real-time polymerase chain reaction; ELISA: enzyme-linked immunosorbent assay; HMGB1: high-mobility group box 1
